# The nervous system of the lophophore in the ctenostome *Amathia gracilis* provides insight into the morphology of ancestral ectoprocts and the monophyly of the lophophorates

**DOI:** 10.1186/s12862-016-0744-7

**Published:** 2016-09-06

**Authors:** Elena N. Temereva, Igor A. Kosevich

**Affiliations:** Department Invertebrate Zoology, Biological Faculty, Moscow State University, Moscow, Russia

**Keywords:** Ectoprocta, Bryozoa, Nervous system, Cerebral ganglion, Tentacles, Lophophore, Lophophorates, Evolution, Phylogeny

## Abstract

**Background:**

The Bryozoa (=Ectoprocta) is a large group of bilaterians that exhibit great variability in the innervation of tentacles and in the organization of the cerebral ganglion. Investigations of bryozoans from different groups may contribute to the reconstruction of the bryozoan nervous system bauplan. A detailed investigation of the polypide nervous system of the ctenostome bryozoan *Amathia gracilis* is reported here.

**Results:**

The cerebral ganglion displays prominent zonality and has at least three zones: proximal, central, and distal. The proximal zone is the most developed and contains two large perikarya giving rise to the tentacle sheath nerves. The neuroepithelial organization of the cerebral ganglion is revealed. The tiny lumen of the cerebral ganglion is represented by narrow spaces between the apical projections of the perikarya of the central zone. The cerebral ganglion gives rise to five groups of main neurite bundles of the lophophore and the tentacle sheath: the circum-oral nerve ring, the lophophoral dorso-lateral nerves, the pharyngeal and visceral neurite bundles, the outer nerve ring, and the tentacle sheath nerves. Serotonin-like immunoreactive nerve system of polypide includes eight large perikarya located between tentacles bases. There are two analmost and six oralmost perikarya with prominent serotonergic “gap” between them. Based on the characteristics of their innervations, the tentacles can be subdivided into two groups: four that are near the anus and six that are near the mouth. Two longitudinal neurite bundles - medio-frontal and abfrontal - extend along each tentacle.

**Conclusion:**

The zonality of the cerebral ganglion, the presence of three commissures, and location of the main nerves emanating from each zone might have caused by directive innervation of the various parts of the body: the tentacles sheath, the lophohpore, and the digestive tract. Two alternative scenarios of bryozoan lophophore evolution are discussed. The arrangement of large serotonin-like immunoreactive perikarya differs from the pattern previously described in ctenostome bryozoans. In accordance with its position relative to the same organs (tentacles, anus, and mouth), the lophophore outer nerve ring corresponds to the brachiopod lower brachial nerve and to the phoronid tentacular nerve ring. The presence of the outer nerve ring makes the lophophore innervation within the group (clade) of lophophorates similar and provides additional morphological evidence of the lophophore homology and monophyly of the lophophorates.

**Electronic supplementary material:**

The online version of this article (doi:10.1186/s12862-016-0744-7) contains supplementary material, which is available to authorized users.

## Background

Bryozoans (= Ectoprocta) are predominantly colonial, benthic freshwater and marine invertebrates. The phylum contains 10,941 species of which 5,455 are extinct [1]. According to other data, the Bryozoa includes over 6,000 extant species and about 15,000 extinct species [2].

The phylogenetic position of bryozoans within bilaterians has yet to be firmly established. Together with phoronids and brachiopods, the bryozoans are traditionally regarded as typical lophophorates because all three groups have a lophophore [3–7]. This traditional view was recently supported by molecular data [8–10] and morphological data [11, 12]. At the same time, many papers regard byozoans as basal lophotrochozoans or group them into the clade Polyzoa, or *etc.* [13–16]. The lophophore looks very similar among all lophophorates but does have peculiarities in each group. For this reason, the investigation of the lophophore and tentacle innervation in additional representative species and especially in “basal” taxa may help clarify the lophophore bauplan.

Investigating the lophophore and tentacle innervation as well as the organization of the cerebral ganglion is also important for understanding the nervous system bauplan within bryozoans. According to recent data, there are three main groups of bryozoans: Phylactolaemata, Stenolaemata, and Gymnolaemata (with the latter comprising the Ctenostomata and Cheilostomata). Many reports have described the innervation of the lophophore and tentacles in many different bryozoans from all of these taxa, except Stenolaemata [2, 17–39]. These data indicate great variation in the organization of the bryozoan nervous system. The variations concern organization of the cerebral ganglion, innervation of tentacles, and connections between tentacular neurite bundles and the main nerve centers. These numerous results were reviewed and analyzed in several recent papers that presented ideas about general patterns of the tentacle innervation and the localization of the lophophore main nerve tracts in all bryozoans [40–42].

Although the Phylactolaemata is the basal group within the bryozoans [43], members of this class occur only in fresh water and apparently acquired some specific and not ancestral features. For this reason, investigation of bryozoans from other groups that may retain ancestral features should provide more insight into the neural bauplan of the Bryozoa in general than investigation of phylactolaemate bryozoans.

A special concern regarding the neural bauplan of the Ectoprocta is the organization of the cerebral ganglion. According to many results, the cerebral ganglion in all bryozoans is formed as an invagination of a portion of epithelium and contains perikarya and neurites [35, 39]. Given this form of development, the cerebral ganglion retains epithelial structure, which has been described in specimens from some groups of bryozoans. In phylactolaemates, the cerebral ganglion contains a central fluid-filled lumen surrounded by a neuroepithelium. The central lumen was recently discovered in certain ctenostome bryozoans [2]. The presence of the lumen in the cerebral ganglion, which disappears during ontogeny in most bryozoans, is regarded as the ancestral state in the Ectoprocta [2].

In this report, we focus on the innervation of the lophophore and tentacles, including the important details about emanations of tentacular neurite bundles, in the ctenostome bryozoan *Amathia gracilis*. This information is essential for determining whether lophophores are homologous within the lophophorates and whether the lophophorates are monophyletic [11]. We also provide a detailed description and analysis of the cerebral ganglion organization in *A. gracilis*, which should help clarify the common organization of the bryozoan ganglion as well as its interconnections with the main nerve tracts.

## Methods

### Sampling of animals and light microscopy

Material was collected near the N.A. Pertsov White Sea Biological Station of Lomonosov Moscow State University (Kandalaksha Bay) (66°34′ N, 33°08′ E). Specimens of *Amathia gracilis* (Leidy, 1855) growing over the bivalve shelves were collected in August 2012 by SCUBA diving at 7–15 m depth. Live animals were photographed using a Panasonic DMC-TZ10 digital camera mounted on a stereo light microscope.

Before fixation, bryozoans were anesthetized overnight in a solution of 5 % MgCl_2_ in fresh sea water (1:1).

### Immunocytochemistry

Animals were fixed with 4 % paraformaldehyde (PFA; Fluka, Germany) in phosphate-buffered saline (PBS; Fluka, Germany) at 4 °C for 24 h. For immunocytochemical staining, the fixed material was rinsed two times for 15 min each time with 3 % BSA (BSA; Sigma, St. Louis, MO, USA) in PBS, and then four times for 1 h each time in a permeabilisation solution containing 0.1 % Triton-×100 (Ferak Berlin, Germany), 0.05 % Tween 20, and 0.1 % NaN_3_ (Sigma) in PBS (PBT). Specimens were then treated with blocking solution (1 % BSA, 0.1 % cold fish skin gelatin (Sigma), 0.5 % Triton X-100, 0.05 % Tween 20, and 0.05 % sodium azide in PBS) (BS) three times for 1 h each time before they were incubated for 36 h at 4 °C with the appropriate primary antibodies diluted in BS. The primary antibodies used were FMRF (rabbit polyclonal, 1:2000; Chemicon, Temecula, CA, USA), anti-serotonin (rabbit polyclonal, 1:1000; Chemicon, Temecula, CA, USA), and anti-tyrosinated α-tubulin (mouse monoclonal, 1:1600; Sigma, USA). After they were washed four times for 3 h each time in BS, the bryozoans were incubated for 36 h at 4 °C with a 1:500 dilution of Donkey Anti-Rabbit IgG Antibodies labeled with Alexa Fluor 546 (Molecular Probes, #A10040) and Donkey Anti-Mouse IgG Antibodies labeled with Alexa FluorR 488 (Molecular Probes, #A21202).

After the material was rinsed three times for 10 min each time in PBS, it was stained with the DAPI nuclei stain (100 ng/ml; Sigma), mounted on a cover glass covered with poly-L-lysine (Sigma-Aldrich, St. Louis, MO, USA), and embedded in Murray Clear, a 2:1 mixture of benzyl benzoate and benzyl alcohol.

Negative controls included specimens processed without incubation in primary antibodies. The autofluorescence control was prepared without addition of fluorochrome (secondary antibodies).

Specimens were examined with a Nikon A1 confocal microscope (Tokyo, Japan) (White Sea Biological Station, Russia) and with a Nikon Eclipse Ti confocal microscope (Tokyo, Japan) (Moscow State University, Moscow, Russia). Optical longitudinal sections were obtained with a 1-μm step size.

Z-projections were generated using the program Image J version 1.43.

### Transmission electron microscopy

For transmission electron microscopy (TEM), the animals were fixed overnight at 4 °C in 2.5 % glutaraldehyde in phosphate buffer saline with addition of NaCl (pH 7.4, osmolarity 830 milliosmols) [44] and were postfixed for 2 h in 1 % osmium tetroxide in the same buffer saline. After the specimens were washed with the same buffer saline, they were transferred through an ethanol series and stored in 70 % ethanol at 4 °C. Further preparation included dehydration in an ethanol series and acetone, and embedding in Epon-Araldite resin (Electron Microscopy Sciences, Fort Washington, PA, USA). Semithin and thin sections were cut with a Ultracut-R Leica ultratome (Leica, Germany). Semithin sections were stained with methylene blue, observed with a Zeiss Axioplan2 microscope, and photographed with an AxioCam HRm camera. Ultrathin sections were stained in uranyl acetate followed by lead nitrate and were examined with JEM-1011 JEOL and JEM-100 B-1 JEOL transmission electron microscopes (JEOL, Akishima, Japan).

Three-dimensional reconstructions were generated using Amira version 5.2.2 software (Bitplane, Zurich, Switzerland). TEM micrographs and Z-projections were processed in Adobe Photoshop (CS3 7.0.1., Adobe Systems) to prepare panoramas and combinations of Z-projections.

### Terminology

The names of the nerve elements are the same as previously used in most papers [2, 27, 32, 41, 42]. Although the term ‘nerve’ was discouraged by Richter and coauthors [45], we use it because it corresponds with the traditional terminology. The specific terminology, which is usually used for description of bryozoans organ systems, is correlated with previous descriptions [2, 32, 41].

## Results

### General morphology of *Amathia gracilis*

**Fig. 1:**
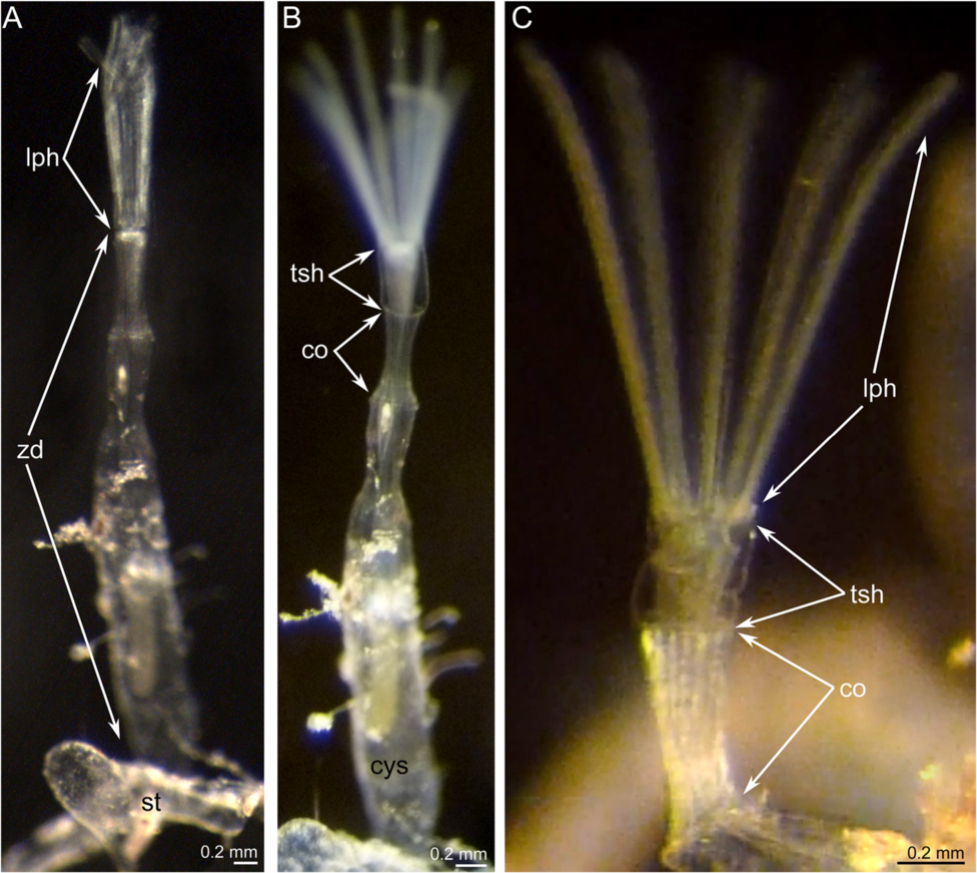
Part of the *Amathia gracilis* colony; photographs of live animals. **a** Zooid with a portion of the stolon. **b** Different parts of the zooid body: cystid, collar, and a tentacle sheath. **c** Anterior part of a zooid: lophophore, tentacle sheath, and collar. The rugosity of the collar is evident. Abbreviations: co - collar; cys - cystid; lph - lophophore; tsh - tentacle sheath; st - stolon; zd - zooid

**Fig. 2:**
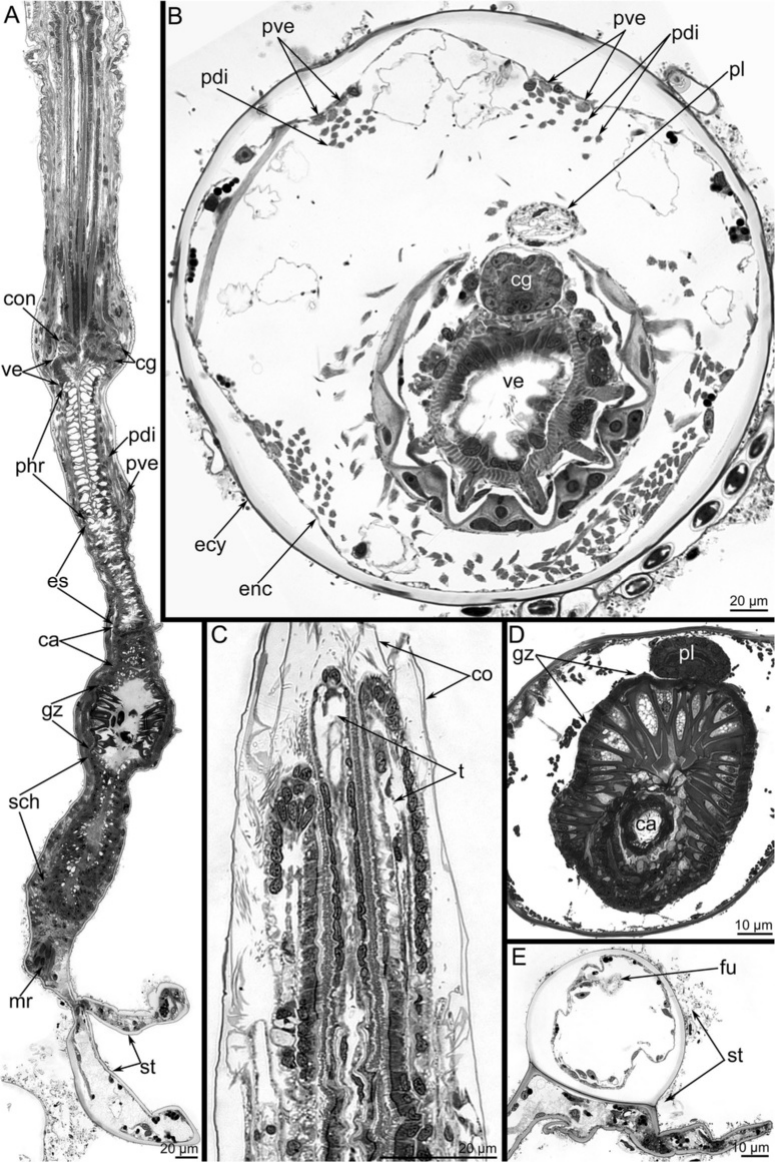
General morphology of *Amathia gracilis* (semithin sections). **a** Longitudinal parasagittal section of the zooid. The oral side is to the left, and the anal side is to the right. The different parts of the digestive tract are shown. **b** Cross section of the cystid at the level of the vestibulum. The polypide is partly retracted into the cystid. The anal side is toward the top, and the oral side is toward the bottom. **c** Longitudinal section of the anterior part of the retracted lophophore, which is covered by the collar (co). **d** Cross section of the gizzard (gz). **e** Cross section of the stolon. The funiculus (fu) is visible. Abbreviations: ca - cardia; cg - cerebral ganglion; co - collar; con - circum - oral nerve ring; ecy - etocyst; enc - endocyst; es - esophagus; mr - muscle-retractor of polypidee; pdi - parietodiaphragmal muscles; phr - pharynx; pl - pylorus; pve - parietovestibular muscles; sch - stomach; st - stolon; t - tentacle; ve - vestibulum

*Amathia gracilis* is a “stolonial” ctenostome: the colony consists of branching stolons divided into internodes, and the tube-like zooids emanating singly from the stolon (Figs. 1a, b and 2a). Each zooid consists of several parts (Fig. 1b, c). The cystid attached to the stolon is the widest and the largest part of the zooid (Fig. 1b) and protects the soft polypide. The cystid is covered by an uncalcified ectocyst, which is an organic noncellular layer produced by the endocyst (Fig. 2b). The upper portion of the ectocyst continues into the thin pleated protrusion that forms a collar (Figs. 1c and 2c). The collar surrounds the lower part of the everted tentacle sheath when the polypide is protruded (Fig. 2c). When the polypide is retracted into the cystid, the collar either remains exposed above the cystid or retracts together with the polypide. The lophophore is the most distal part of the zooid. The lophophore bears 10 tentacles, which are covered by cilia and which function in food capture. The lophophore with the tentacle sheath and the digestive tract form a polypide.

As in other bryozoans, the digestive tract in *A. gracilis* is U-shaped. Because of this morphology, the mouth and anus are located near each other. The side contiguous with the mouth is called the oral side; the opposite side nearest to the anus is called the anal side. The digestive tract consists of vestibulum (the portion between the mouth and the pharynx), pharynx, cardia, gizzard, stomach with caecum, and pylorus followed by the rectum (Fig. 2a, d). Special retractor muscles retract the polypide into the cystid (Fig. 2a). Other longitudinal muscles-the parieto-vestibular and parieto-diaphragmal muscles-extend parallel to each other along the walls of the cystid in its upper part (Fig. 2b). The funiculus is the thin “mesentery” that passes from the caecum of each zooid to the stolon (Fig. 2e).

### Cerebral ganglion

**Fig. 3:**
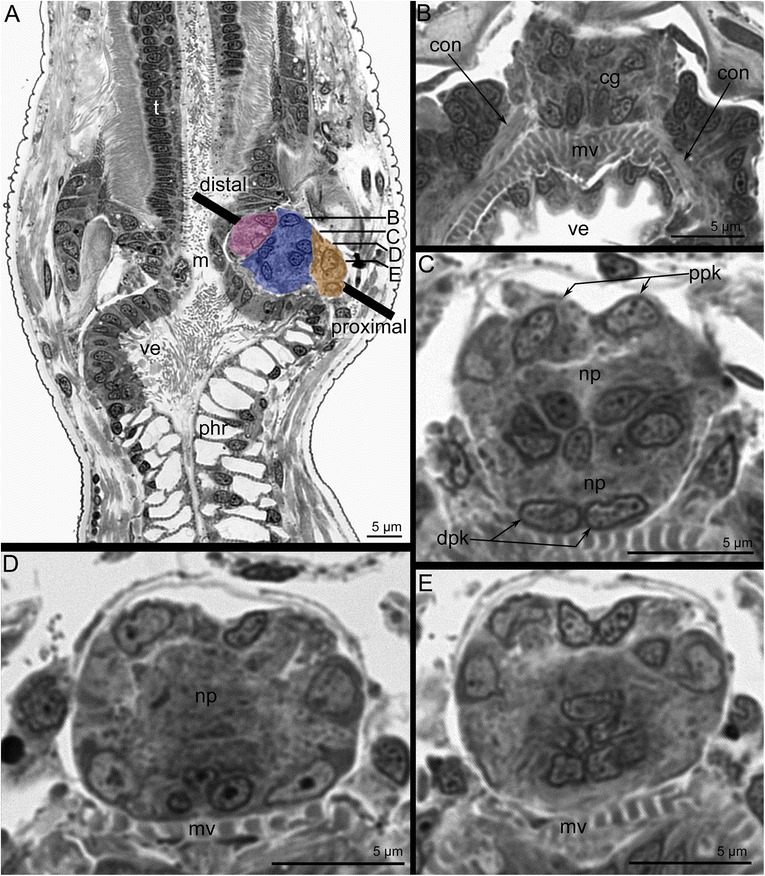
Organization of the cerebral ganglion of *Amathia gracilis* (semithin sections). **a** Longitudinal section of the cerebral ganglion in the zooid. The colours mark different zones of the ganglion: pink - oral (distal), blue - central, yellow - anal (proximal). The oral side is to the left, and the anal side is to the right. The thick black line indicates the disto-proximal axis. **b**–**e** Cross sections of the cerebral ganglion at different levels (shown on A). The anal side is toward the top, and the oral side is toward the bottom. **b** The uppermost portion of the ganglion. Two zones are evident. **c** 4 μm below the section in Fig. B. **d** 3 μm below the section in Figure C. **e** 3 μm below the section in Figure D. Abbreviations: cg - cerebral ganglion; con - circum - oral nerve ring; dpk - pair of distal perikarya; m - mouth; mv - muscles of vestibulum; phr - pharynx; np - neuropil; ve - vestibulum; ppk - pair of proximal perikarya

**Fig. 4:**
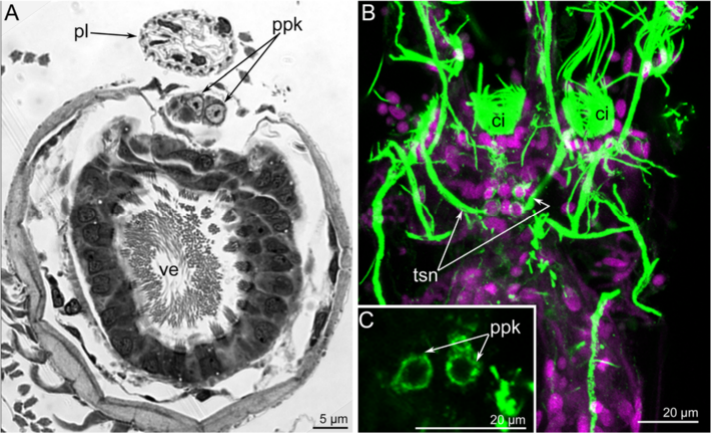
Organization of the proximal zone of the cerebral ganglion of *Amathia gracilis*. **a** Transverse semithin section of the proximal zone of the cerebral ganglion. The anal side is toward the top, and the oral side is toward the bottom. **b** The lophophore base: confocal sections (LCM). Z-stack projection of several slides adjacent to the anus after double staining for tyrosinated α-tubulin (green) and DAPI (magenta). **c** LCM: Z-projection of two most distal perikarya. Abbreviations: ci - cilia of tentacles; pl - pylorus; ppk - proximal perikarya; tsn - tentacle sheath nerve; ve - vestibulum

The cerebral ganglion is located at the base of the lophophore, on the anal side of the vestibulum (Figs. 2a, b and 3). It is a compact ovoid structure elongated in the oral-anal direction (17.5 μm) and flattened in the zooid longitudinal direction (11 μm) (Figs. 3a and 4a).

**Fig. 5:**
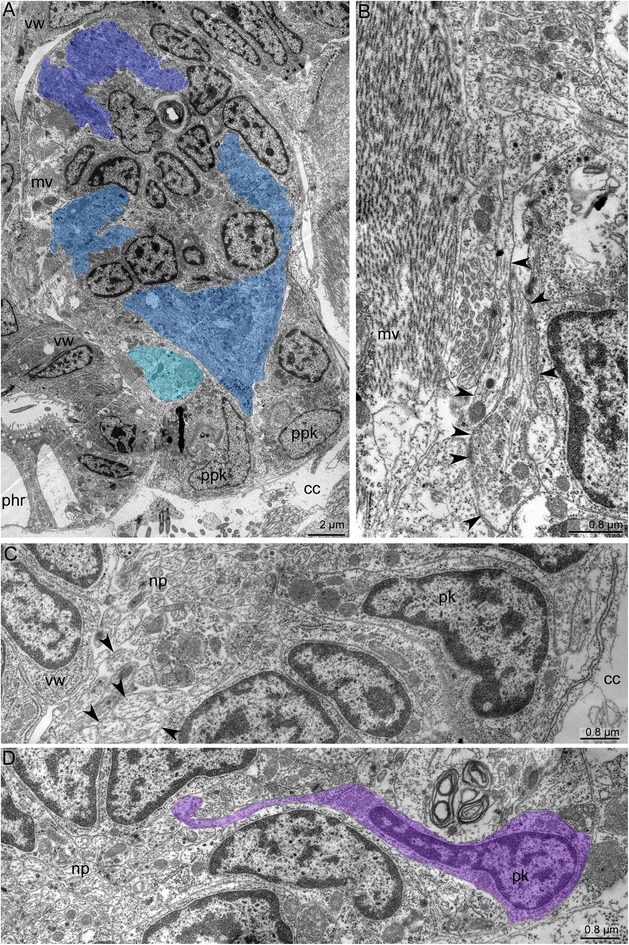
Ultrastructure of the cerebral ganglion and circum-oral nerve ring in *Amathia gracilis*. **a** Longitudinal section of the cerebral ganglion. The oral side is to the upper left corner, and the anal side is to the lower right corner. The color indicates distal (violet), central (blue), and proximal (cyan) neuropiles. **b** Part of the proximal neuropil of the cerebral ganglion. Arrowheads indicate the neurites of large diameters and with thick microtubules. **c** Part of the circum-oral nerve ring, including the perikarya and the neuropil with neurites of large diameter (arrowheads). **d** Part of the circum-oral nerve ring with the perikaryon (magenta) and the apical projection extending into the neuropil. Abbreviations: cc - coelomic cavity; mv - muscles of vestibulum; np - neuropil; phr - pharynx; pk - perikaryon; ppk - proximal perikarya; vw - wall of vestibulum

The cerebral ganglion has zones consisting of several groups of perikarya and neurite bundles (Fig. 3). In longitudinal sections, three zones, each formed by a compact group of perikarya, can be recognized in the cerebral ganglion (Figs. 3a and 4a). These zones are separated from each other by several neuropiles (Figs. 4a and 5a). In cross sections that pass obliquely to the oral-anal axis of the ganglion, the zonality is not evident, and the zonality is absent in the uppermost sections of the cerebral ganglion (Fig. 3b). At middle levels, the zonality seems to be tripartite, and each zone appears to have bilateral symmetry and is formed by paired perikarya (Fig. 3c–e).

In cross and longitudinal sections of the cerebral ganglion, the most prominent zone is the anal (proximal) one, which is formed by paired, large perikarya (Fig. 4a–e). These perikarya have large nuclei with electron-lucent karyoplasm and a prominent nucleolus (Fig. 4a–b). Two most proximal perikarya give rise to the thick longitudinal neurite bundles of the tentacle sheath (Fig. 4c–d).

**Fig. 6:**
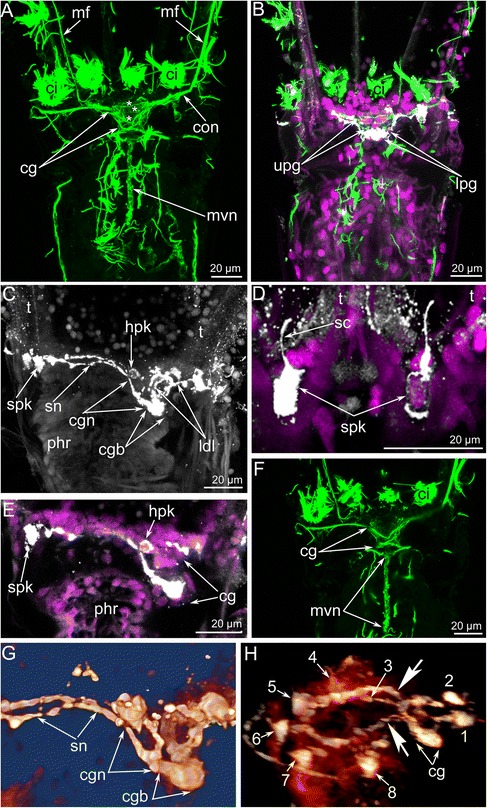
Some details of the cerebral ganglion and visceral nerve organization in *Amathia gracilis*. Z-projections (**a**–**f**) of the lophophore after mono-, double, and triple staining for tyrosinated α-tubulin (green), 5-HT (serotonin) (white), and DAPI (magenta). **a** Anal view of the cerebral ganglion. Three commissures are indicated by asterisks. **b** Anal view of the central zone of the cerebral ganglion, which consists of upper and lower parts. **c** Serotonin-like immunoreactive nerve elements within the lophophore. Lateral view; the oral side is to the left, and the anal side is to the right. **d** Serotonin-like immunoreactive perikarya located at the base of the lophophore between tentacle bases. **e** Some details of the ganglion organization: ganglion ventro-lateral nerves are associated with large multipolar perikarya. Lateral view. **f** Medial visceral nerve starting from the cerebral ganglion. **g** 3D reconstruction of the serotonin-like innunoreactive parts of the cerebral ganglion and the circum-oral nerve ring including the intertentacular neurite bundles. **h** Volume rendering of the serotonin-like immunoreactive nervous system. The anal side is to the right; the oral side is to the left. Numbers 1-8 indicate serotonin-like immunoreactive perikarya. Large arrows indicate the “gaps” between two analmost and six oralmost perikarya. Abbreviations: ci - cilia of tentacles; cg - cerebral ganglion; cgb - proximal part of the cerebral ganglion; cgn - ventro-lateral nerves of the cerebral ganglion; con - circum-oral nerve ring; hpk - perikaryon of the cerebral ganglion ventro-lateral nerve; ldl - lophophoral dorso-lateral nerves; lpg - lower portion of the cerebral ganglion; mf - medio-frontal nerve of tentacle; mvn - medial visceral nerve; phr - pharynx; sc - serotonincilia; sn - serotonin-like immunoreactive intertentacular neurites; spk - serotonin-like immunoreactive perikarya between tentacles; t - tentacle; upg - upper portion of the cerebral ganglion

**Fig. 7:**
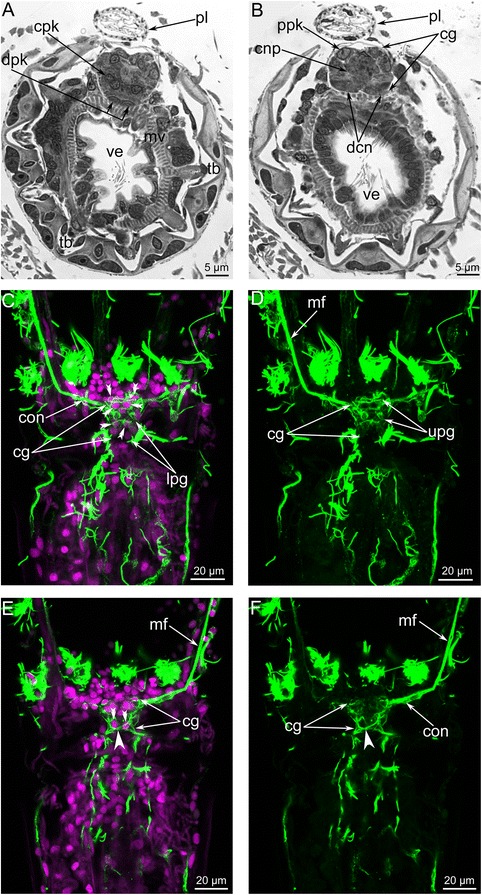
Organization of the central zone of the cerebral ganglion in *Amathia gracilis*. Semithin cross sections (**a**–**b**): the anal side is at the top, and the oral side is at the bottom. Anal view of Z-projections (**c**–**f**) of the lophophore after mono- and double staining for tyrosinated α-tubulin (green) and DAPI (magenta). **a** Cross section at the level of the tentacle bases. **b** Cross section at the level of the vestibulum. **c** Paired perikarya (double arrowheads) in the central zone of the cerebral ganglion. **d** Neurites of the central zone of the cerebral ganglion. **e** Paired perikarya (double arrowheads) and the chiasm (arrowhead) in the lower portion of the central zone. **f** Neurites forming a chiasm (arrowhead) in the central zone. Abbreviations: cg - cerebral ganglion; cnp - neuropil of central zone; cpk - perikarya of central zone; con - circum-oral nerve ring; dcn - cross neuropiles (commissures) in the distal zone; dpk - perikarya of distal zone; lpg - lower portion of the cerebral ganglion; mf - medio-frontal nerve of tentacle; mv - muscles of vestibulum; pl - pylorus; ppk - perikarya of proximal zone; tb - tentacle base; ve - vestibulum; upg - upper portion of the cerebral ganglion

**Fig. 8:**
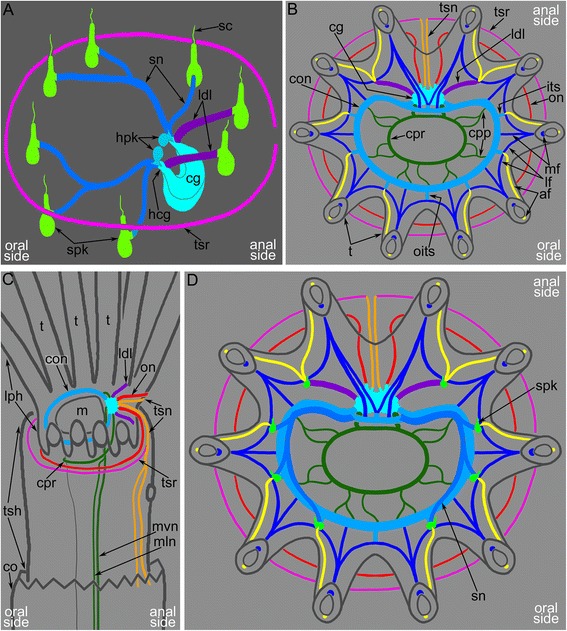
Schemes of the lophophore and of the tentacle sheath innervation in *Amathia gracilis*. **a** Organization of the serotonin-like immunoreactive nervous system of the lophophore, viewed from the left. **b** Top view of the nerve elements after staining for tyrosinated α-tubulin. **c** The main nerve tracts of the lophophore and the tentacle sheath. Lateral view. Only the bases of the front tentacles are shown. **d** Combination of the serotonin-like and the α-tubulin-like immunoreactive nerve elements of the lophophore. Top view. Abbreviations: af - abfrontal tentacle nerve; cg - cerebral ganglion; co - collar; con - circum-oral nerve ring; cpr - circum-pharyngeal nerve ring; cpp - circum-pharyngeal nerve plexus; cgn - ventro-lateral nerves of the cerebral ganglion; hpk - perikaryon of the cerebral ganglion ventro-lateral nerve; its - intertentacular site; ldl - lophophoral dorso-lateral nerve; lf - latero-frontal tentacle nerve; lph - lophophore; m - mouth; mf - medio-frontal tentacle nerve; mln - medio-lateral visceral nerve; mvn - medial visceral nerve; oits - intertentacular site closest to the mouth; on - outer nerve ring; sc - serotonincilia; sn - serotonin-like immunoreactive intertentacular neurites; spk - serotonin-like immunoreactive perikarya between tentacles; t - tentacle; tsh - tentacle sheath; tsn - tentacle sheath nerve; tsr - tentacle sheath nerve ring

**Fig. 9:**
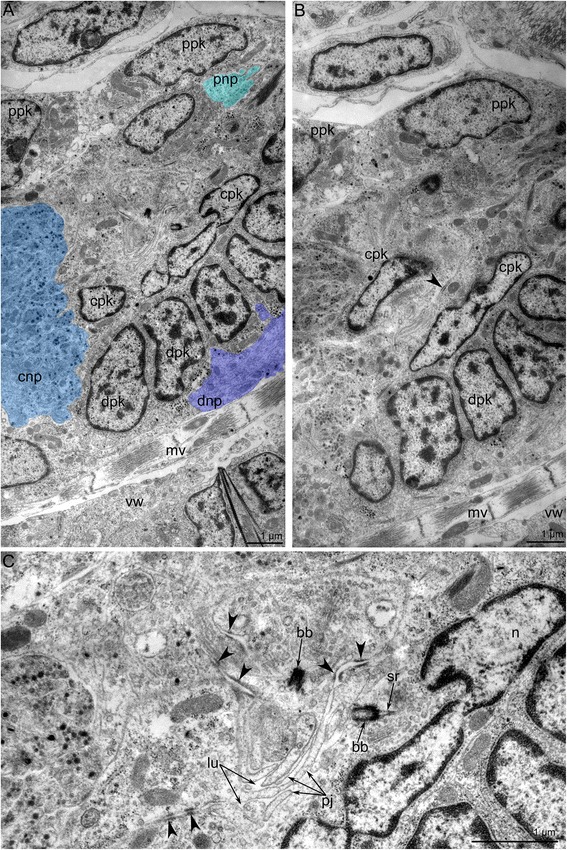
Ultrastructure of the cerebral ganglion in *Amathia gracilis*. Ultrathin cross sections; the anal side is toward the top, and the oral side is toward the bottom. **a** Three neuropiles of the cerebral ganglion marked by colors: proximal - cyan, central - blue, distal - violet. **b** Overview of all zones of the cerebral ganglion: perikarya of proximal, central, and distal zones. **c** Portion of the central zone - neuroepithelium. The lumen of the cerebral ganglion is filled with apical projections of the nerve cells, which connect via desmosomes (arrowheads) and bear the basal body. Abbreviations: bb - basal body; cnp - central neuropil; cpk - perikarya of central zone; dnp - distal neuropil; dpk - perikarya of distal zone; lu - lumen of cerebral ganglion; mv - muscle of vestibulum; n - nucleus; sr - striated rootlet; pj - apical projections; pnp - proximal neuropil; ppk - perikarya of proximal zone; vw - wall of vestibulum

The central zone of the ganglion has a complex structure and subdivided into the distal and the proximal parts (Fig. 6a, b). Each part includes a voluminous central neuropil and perikarya (Figs. 3 and 7a, b). The lower part exhibits serotonin-like immunoreactivity and gives rise to the thin outer nerve and serotonin-like immunoreactive intertentacular neurites (Figs. 6b and 8a). The serotonin-like immunoreactive portion of the cerebral ganglion has a horseshoe-like shape with two ventro-lateral nerves (Figs. 6c, g and 8a). Each nerve is associated with a large multipolar serotonin-like immunoreactive perikaryon (Figs. 6c, e and 8a). The upper portion of the central zone gives rise to the dorso-lateral nerves of the lophophore, the circum-oral nerve ring, and the nerve plexus of the pharynx (Figs. 7c–f and 8b, c). According to the immunocytochemical data, the central zone of the cerebral ganglion includes perikarya with neurites that form a chiasma (Fig. 7e, f). TEM data revealed the presence of numerous electron-dense granules in the cytoplasm of the perikarya of the central zone (Fig. 9a).

The zone closest to the mouth (the distal zone) of the cerebral ganglion includes paired perikarya with small elongated nuclei (Figs. 3c and 7a) and paired transverse neurite bundles (Fig. 7b). The distal zone contributes to the innervation of tentacles and to the formation of the circum-oral nerve ring.

The cerebral ganglion lacks a voluminous lumen (Figs. 3b–e and 7a, b). Although a clear space exists between the proximal and central zones of the cerebral ganglion, it is not a true lumen (Fig. 3c–e). This space is filled with apical projections of the perikarya (Fig. 9b, c). This portion of the cerebral ganglion exhibits characteristics of neuroepithelium. Perikarya contact each other via several desmosomes (Fig. 9b, c). Some perikarya bear basal bodies with short, striated rootlets (Fig. 9c).

The cerebral ganglion contains several neuropiles (Figs. 5a and 9a). The distal neuropil is associated with distal perikarya and contacts the wall of the vestibulum. The central neuropil occupies the largest spece within the cerebral ganglion. Neurites of the central neuropil contain electron-dense granules (Fig. 9a). The proximal neuropil is associated with the proximal perikarya. The proximal neurites have large diameters and contain many thick microtubules; the diameter of these microtubules is about 22 nm (Fig. 5b).

**Fig. 10:**
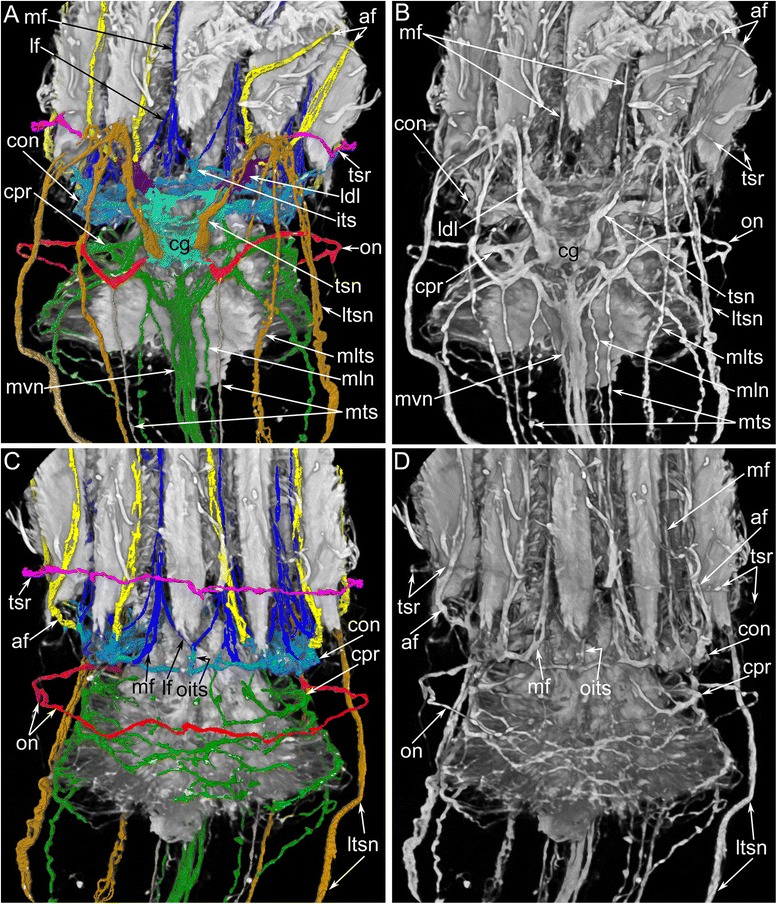
Reconstructions of the nervous system of the lophophore and tentacle sheath in *Amathia gracilis*. 3D-reconstructions (**a**, **c**) combined with volume renderings based on confocal image stacks. Volume renderings (**b**, **d**). Staining with antibodies against tyrosinated α-tubulin. Ends of the tentacles are on the top. **a**–**b** Anal view of the lophophore and the tentacle sheath. **c**–**d** Oral view of the lophophore and the tentacle sheath. Abbreviations: af - abfrontal tentacle nerve; cg - cerebral ganglion; con - circum-oral nerve ring; cpr - circum-pharyngeal nerve ring; its - intertentacular site; ldl - lophophoral dorso-lateral nerve; lf - latero-frontal tentacle nerve; ltsn - lateral tentacle sheath nerve; mf - medio-frontal tentacle nerve; mln - medio-lateral visceral nerve; mlts - medio-lateral tentacle sheath nerve; mts - medial tentacle sheath nerve; mvn - medial visceral nerve; oits - intertentacular site closest to the mouth; on - outer nerve ring; tsn - tentacle sheath nerve; tsr - tentacle sheath nerve ring

**Fig. 11:**
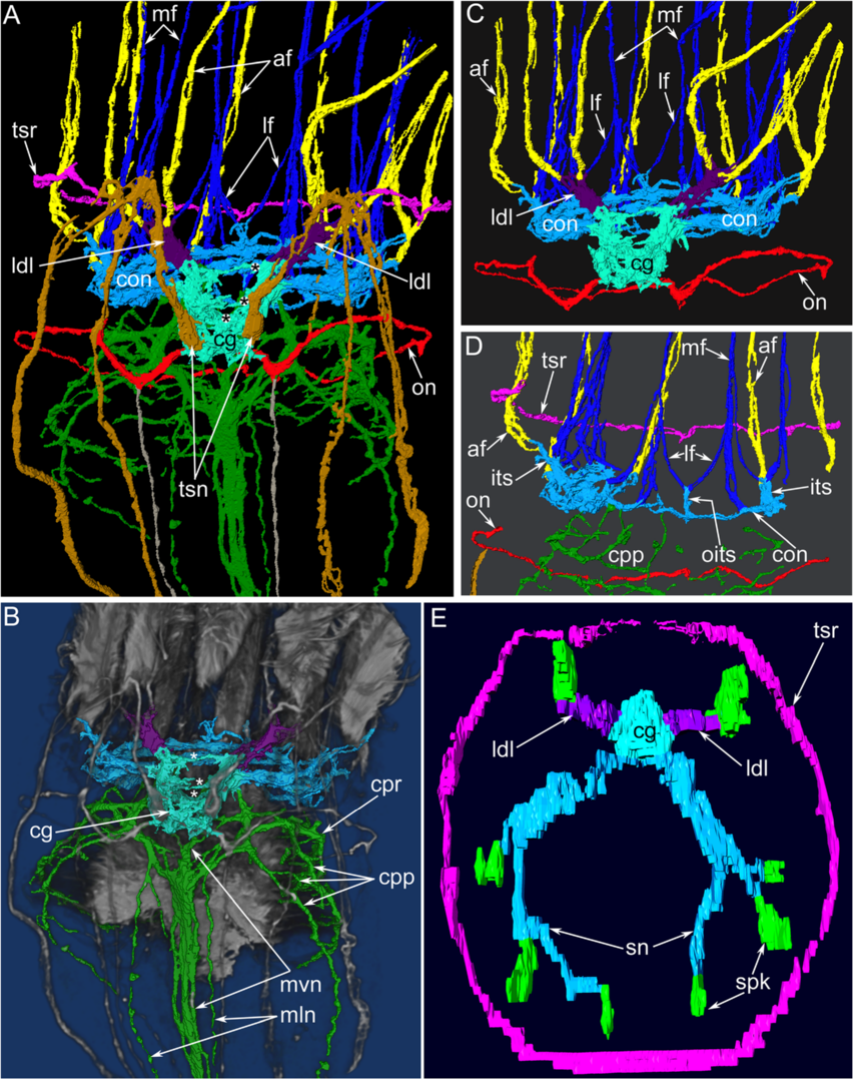
Details of innervation of the lophophore and the tentacles in *Amathia gracilis*. 3D-reconstructions (**a**, **c**–**e**) and 3D-reconstructions combined with volume renderings based on confocal image stacks (**b**). The apical is on the top in all micrographs except for **E**, which is viewed from the top of the lophophore. **a** Main nerve tracts projecting from the cerebral ganglion. Anal view. Three commissures of the cerebral ganglion are indicated by asterisks. **b** Localization of the circum-pharyngeal nerve plexus and visceral nerve tracts. Three commissures of the cerebral ganglion are indicated by asterisks. **c** Tentacular nerves projecting from the circum-oral nerve ring and lophophoral dorso-lateral nerves. **d** Innervation of the tentacles closest to the mouth: the intertentacular site closest to the mouth has no connection to the abfrontal tentacle nerve. View from the anal side. **e** 3D-reconstruction of the serotonin-like immunoreactive nerve system of the lophophore. Abbreviations: af - abfrontal tentacle nerve; cg - cerebral ganglion; con - circum-oral nerve ring; cpp - circum-pharyngeal nerve plexus; cpr - circum-pharyngeal nerve ring; its - intertentacular site; ldl - lophophoral dorso-lateral nerve; lf - latero-frontal tentacle nerve; mf - medio-frontal tentacle nerve; mln - medio-lateral visceral nerve; mvn - medial visceral nerve; oits - intertentacular site closest to the mouth; on - outer nerve ring; sn - serotonin-like immunoreactive intertentacular neurites; spk - serotonin-like immunoreactive perikarya between tentacles; tsn - tentacle sheath nerve; tsr - tentacle sheath nerve ring

According to 3D-reconstructions, the cerebral ganglion contains several cross commissures (Figs. 6a, 10a and 11a–c). The distal commissure corresponds to the outbranching of the lophophoral dorso-lateral nerves. The middle commissure is located where the ganglion connects to the circum-oral nerve ring. The proximal commissure is located within the most proximal portion of the ganglion.

### Nerves projecting from the cerebral ganglion

The cerebral ganglion gives rise to several main neurite bundles that contribute to the innervation of the lophophore with tentacles, the tentacle sheath, and the digestive tract. Five pairs of main nerve tracts arising from the cerebral ganglion can be recognized: the circum-oral nerve ring, the lophophoral dorso-lateral nerves, the pharyngeal and the visceral neurite bundles, the outer nerve ring, and the tentacle sheath nerves (Figs. 8b, c, 10a–d and 11a–c).

The circum-oral nerve ring arises from the cerebral ganglion and continues around the vestibulum. The circum-oral nerve ring includes six serotonin-like immunoreactive neurites starting from the cerebral ganglion and extending to the sites between the six oral tentacles (Figs. 8a, d and 11e). These neurites do not form a closed circle around the vestibulum; thus, the serotonin-like immunoreactive portion of the circum-oral nerve ring is horseshoe shaped (Figs. 8a and 11e). Each of the six serotonin-like immunoreactive neurites is associated with a large cell that exhibits strong serotonin-like immunoreactivity. These cells are located at the sites between the tentacles (Fig. 8d). Each cell bears a cilium and is apparently sensory (Figs. 6d and 8a). In *A. gracilis*, there are eight serotonin-like immunoreactive sensory perikarya between the tentacles bases: six perikarya are associated with the circum-oral nerve ring, and two perikarya are connected to the lophophoral dorso-lateral neurite bundles (Figs. 6h, 8a, d and 11e). There are two “gaps” between two analmost and six oralmost serotonin-like immunoreactive pekirarya (Additional file 1, Fig. 6h).

The circum-oral nerve ring includes neurites and perikarya (Fig. 5c, d). The perikarya are located on the periphery and give rise to apical projections that contribute to the neuropil of the circum-oral nerve ring (Fig. 5d). Two types of neurites can be recognised within the neuropil based on the differences in their diameters. Large neurites have electron-lucent cytoplasm containing thick microtubules, whereas small neurites have electron-dense cytoplasm that usually contains some granules and mitochondria. Within the neuropil, the large neurites occupy in the proximal region (Fig. 5c).

**Fig. 12:**
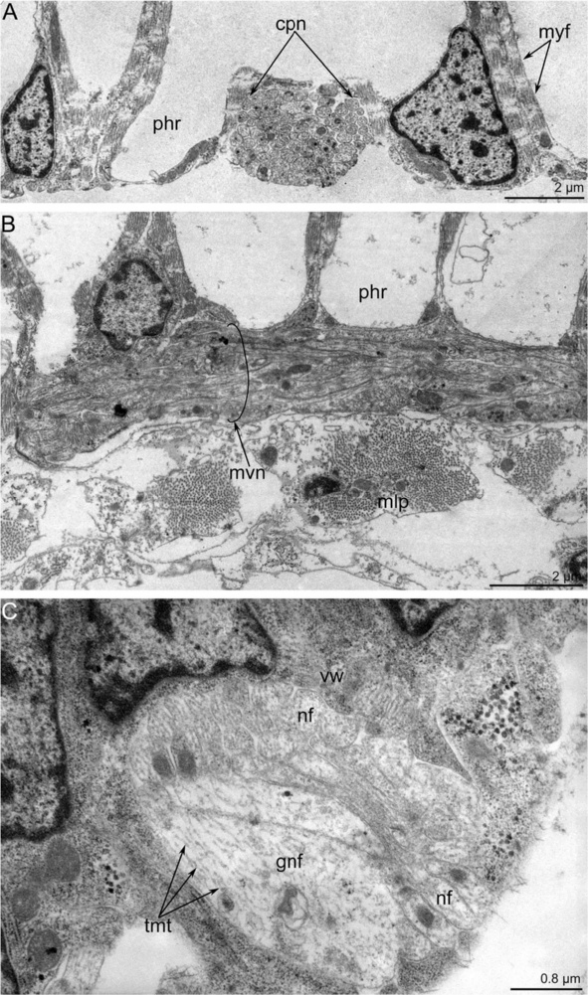
Innervation of the digestive tract in *Amathia gracilis*. Longitudinal **a**–**b** and transversal (**c**) ultrathin sections. **a** Portion of the pharynx wall with the circular neurite bundles. **b** Medial visceral nerve (in the circle). **c** Neurite bundle in the vestibulum wall contains giant neurites and nerve fibers of common diameter. Abbreviations: cpn - circum-pharyngeal neurite bundle; gnf - giant nerve fiber; mlp - muscular lining of pharynx; myf - myofilaments; mvn - medial visceral nerve; nf - nerve fibers; phr - pharynx; tmt - thick microtubules; vw - wall of vestibulum

The pharyngeal nerve plexus begins as the circum-pharyngeal nerve ring and gives rise to the numerous circular and longitudinal neurite bundles (Figs. 11b and 12a–b). These neurite bundles extend towards the esophagus. The wall of the vestibulum contains some longitudinal neurite bundles that consist of several neurites including the giant nerve fibers (Fig. 12c). These nerve fibers are about 1.5 μm in diameter, which is several times the diameter of most other neurites within the bundle.

The medial visceral nerve is the thick neurite bundle starting from the cerebral ganglion and extending along the anal side of the pharynx and esophagus (Figs. 6f, 10a–b and 11b). The medial visceral nerve is about 2 μm in diameter and consists of several groups of longitudinal neurites (Fig. 12b). The medial visceral nerve gives rise to a pair of medio-lateral visceral neurite bundles and a pair of latero-visceral neurite bundles (Figs. 10a–b and 11b).

**Fig. 13:**
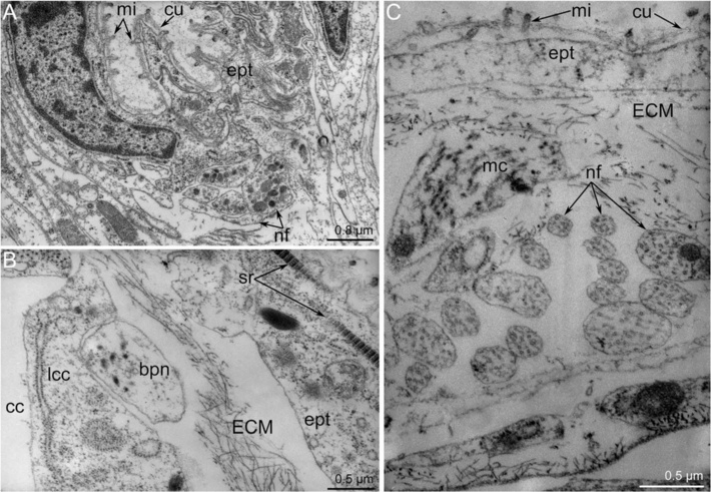
Ultrastructure of certain nerve elements in *Amathia gracilis*. Longitudinal (**a**) and transverse (**b**–**c**) ultrathin sections. **a** Outer nerve ring. **b** Basiperitoneal neurite on the lateral side of the tentacle. **c** Tentacle sheath nerve. Abbreviations: bpn - basiperitoneal neurite; cc - coelomic cavity; cu - cuticle; ECM - extracellular matrix; ept - epithelium; lcc - lining of coelomic cavity; mc - muscle cell; mi - microvilli; nf - nerve fibers; sr - striated rootlets

The outer nerve ring of the lophophore starts from the proximal zone of the cerebral ganglion and extends around the outer side of the lophophore base (Figs. 10a–c and 11a-c). The outer nerve ring of the lophophore is associated with a pair of longitudinal medial nerves of the tentacle sheath. According to TEM data, the outer nerve of the lophophore consists of 3-5 neurites that contain mitochondria, large vesicles with electron-dense content, and small electron-lucent vesicles (Fig. 13a).

The tentacle sheath nerves are the paired neurite bundles projecting from the proximal perikarya of the cerebral ganglion (Figs. 10a–b and 11a). Each nerve starts as a thick neurite bundle branching at the apex of the tentacle sheath and gives rise to the lateral and medio-lateral longitudinal nerves of the tentacle sheath. Each longitudinal nerve consists of 10–15 neurites with electron-lucent cytoplasm and thick microtubules (Fig. 13b). Both of the thick nerves of the tentacle sheath give rise to the circular nerve that extends around the tip of the tentacle sheath (Figs. 10c-d and 11e). This circular nerve shows strong serotonin-like immunoreactivity.

### Innervation of tentacles

**Fig. 14:**
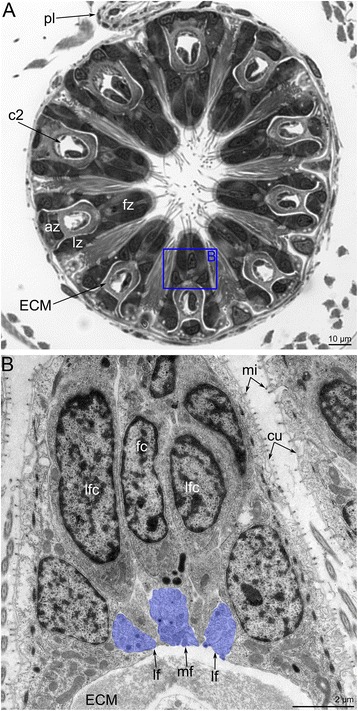
Organization of tentacles in *Amathia gracilis*. **a** Semithin transverse section of tentacle base. Zonality of the tentacle epithelium is evident. **b** Ultrathin transverse section of the frontal and latero-frontal zone of the tentacle base. Abbreviations: az - abfrontal zone; cc - coelomic cavity; cu - cuticle; ECM - extracellular matrix; fc - frontal cell; fz - frontal zone; lf - latero-frontal tentacle nerve; lfc - latero-frontal cell; lz - lateral zone; mi - microvilli; mf - medio-frontal tentacle nerve; pl - pylorus

*Amathia gracilis* has 10 tentacles, each exhibiting zonality of the epithelium in cross section (Fig. 14a). Cells of frontal and latero-flontal zones face the mouth and form a thick epithelium. The lateral zones consist of flattened cells with numerous cilia and numerous long striated rootlets. The abfrontal zone is opposite of the mouth and is formed by flattened nonciliated cells.

According to the pattern of innervation, the 10 tentacles of *A. gracilis* can be subdivided into two groups: four that are near the anus and six that are near the mouth (Fig. 8d). Innervation of four anal tentacles originates from lophophoral dorso-lateral nerves and the anal part of the circum-oral nerve ring. The six oral tentacles are innervated from the radial nerves (= the intertentacular sites), which extend from the circum-oral nerve ring.

**Fig. 15:**
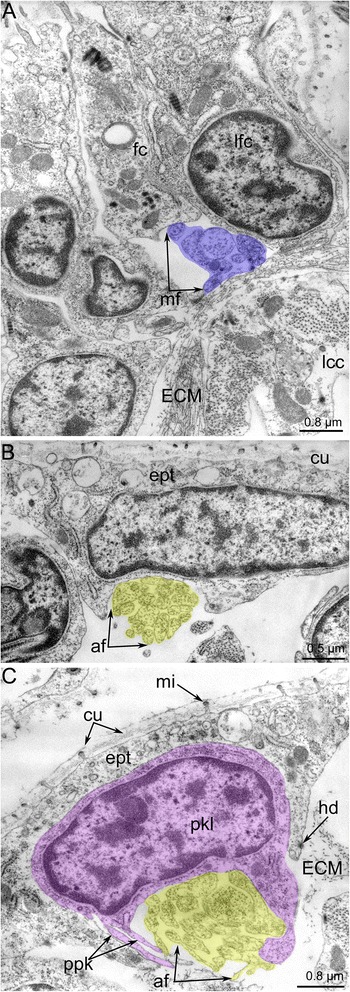
Innervation of tentacles in *Amathia gracilis*. Ultrathin transverse sections of the middle part of tentacles. **a** Frontal zone with the medio-frontal tentacle nerve. **b** Abfrontal zone with the abfrontal neurite bundle. **c** Abfrontal neurite bundle associated with the large perikaryon-like cell. Abbreviations: af - abfrontal neurite bundle; cu - cuticle; ECM - extracellular matrix; ept - epithelium; hd - hemidesmosoma; lcc - lining of coelomic cavity; mf - medio-frontal tentacle nerve; mi - microvilli; pkl - perikaryon-like cell; ppk - projections of perikaryon-like cell

In both the anal and oral tentacles, the circum-oral nerve ring gives rise to the medio-frontal neurites of each tentacle (Fig. 8b, d). The latero-frontal nerves originate from the adjacent intertentacular sites. In the case of anal tentacles, one latero-frontal nerve within each of the four tentacles arises from the intertentacular site of the circum-oral nerve ring, whereas the second latero-frontal nerve originates from the lophophoral dorso-lateral nerve. At the base of each tentacle, there are three frontal neurite bundles: one medial and two lateral (Fig. 14b). These neurite bundles fuse at the base of each tentacle, forming a single frontal neurite bundle extending along the frontal side of the tentacle. Each frontal neurite bundle consists of a few neurites (8–10) extending between the epidermal cells (Fig. 15a).

Each tentacle bears a single abfrontal nerve starting either from the intertentacular sites in the six oral tentacles or from the lophophoral dorso-lateral nerves in the four anal tentacles (Fig. 11c). The intertentacular site near the mouth is not associated with the abfrontal nerve (Fig. 11d). The abfrontal nerve consists of numerous neurites (12–16) with electron-lucent cytoplasm and thick microtubules (Fig. 15b). The abfrontal nerve is associated with the perikarya, whose projections extend into the nerve (Fig. 15c).

There are two basiperitoneal nerve tracts extending along the lateral sides of every tentacle. Each nerve tract consists of a single neurite of large diameter (Fig. 13c).

## Discussion

### The structure of the cerebral ganglion

The cerebral ganglion is the main nerve centre in all bryozoans. Its shape varies among bryozoan groups: it is a vesicle in the phylactolaemates and an ovoid compact mass in most gymnolaemates [41]. The diameter of the cerebral ganglion usually ranges from 30 μm in stoloniferan ctenostomes to 60 μm in “ascophoran” cheilostomes [32]. In *Amathia gracilis*, the diameter of the cerebral ganglion along its oral-anal axis is < 17 μm. It is the smallest ganglion reported thus far for a ctenostome bryozoan.

The zonality of the cerebral ganglion has been described in phylactolaemates and cheilostomates. In phylactolaemates, the basal portion of the cerebral ganglion includes three lobes: one central mass and two lateral masses [32, 35]. At the same time, the ganglion of phylactolaemates exhibits heterogeneity in the oral-anal direction: the oral portion of the ganglion is formed by thin epithelium, while the anal portion is formed by thick epithelium [41]. The zonality of the cerebral ganglion in the cheilostome *Electra pilosa* is expressed in an apical-basal direction [26, 27]. Lutaud has described three zones: distal, central, and proximal. The distal (the upper most) zone gives rise to the circumpharyngeal nerve ring. The central zone contains a complex of three rows of cells, including a large multipolar central cell connecting to both the distal and proximal regions. The proximal zone includes “giant” perikarya that give rise to the tentacle sheath nerves [26, 27].

The cerebral ganglion zonality was not previously described in ctenostomes [2]. In the present paper, the zonality of the cerebral ganglion in ctenostomes is described for the first time. In *A. gracilis*, the cerebral ganglion exhibits prominent zonality along the oral-anal axis. At first glance, this zonality appears to differ from that of other cheilostomes. The cerebral ganglion of *A. gracilis* adjoins tightly with the mouth and vestibulum wall above the beginning of the pharynx, which has a large outer diameter (Fig. 3a). This causes inclination of the cerebral ganglion longitudinal axis in correspondence with the longitudinal axis of the zooid. Still, the cerebral ganglion longitudinal axis is about parallel with the oral-anal direction and can be compared with the disto-proximal axis in *E. pilosa* [26, 27]. If that inference is correct, the cerebral ganglion zonation described in both species is similar. The proximal zone of the cerebral ganglion in *E. pilosa* corresponds to the anal (proximal) zone of *A. gracilis*. In both species, the giant perikarya are located exactly in the proximal zone. The central zone of the cerebral ganglion in *E. pilosa* corresponds to the central zone in *A. gracilis*. In the latter species, the central zone includes some perikarya, whose processes form a chiasm. The presence of a chiasm confirms the integral function of the central zone of the cerebral ganglion. The zonation reflected in the architecture of the cerebral ganglion of some bryozoans apparently provides more directive innervation of the various body parts (lophophore, tentacle sheath, digestive tract, etc.). Such sort of specialization of the central nerve system is known for many other invertebrates [46].

In *A. gracilis*, the cerebral ganglion contains three cross commissures that extend transversally relative to the oral-anal axis. This direction corresponds to the anterior-posterior axis of bilaterians that retain the ancestral body plan (with the worm-like body and the dorsal side equal to the ventral one). The same commissures are found in the cerebral ganglion of *Cristatella mucedo* and *Plumatella repens* [40]. According to several authors, the presence of these commissures could be associated with the coordinated activities of the lophophore arms in these species [47–49].

**Fig. 16:**
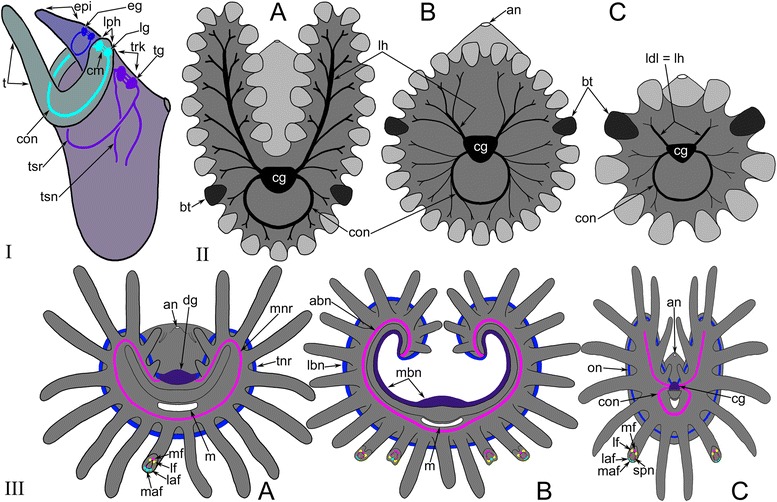
Schemes of several hypothetical steps of bryozoan evolution. **I**) Hypothetical bryozoan ancestor with three body parts: epistome, lophophore with tentacles, and the trunk. Each part has paired ganglion. The cerebral ganglion of recent bryozoans forms as a result of aggregation of these three paired ganglia. **II**) Possible scenario of evolution of the bryozoan lophophore. Both directions of evolution are correct. **a** Horseshoe-shaped lophophore known in some recent phylactolaemates. **b** Bell-shaped lophophore with numerous tentacles. **c** Lophophore of *A. gracilis*. **III**) Gross anatomy of the nervous system of the lophophore in the lophophorates: phoronids (**a**), brachiopods (**b**), and bryozoans (**c**). The hypothetical bryozoan ancestor had a horseshoe-shaped lophophore, six tentacular nerves, and an outer nerve. Abbreviations: abn - accessory brachial nerve; an - anus; bt - “border” (“frontier”) tentacle; cg - cerebral ganglion; cm - commissure; con - circum-oral nerve ring; dg - dorsal ganglion; eg - epistomal ganglion corresponds to distal zone of cerebral ganglion; epi - epistome; laf - latero-abfrontal nerve of tentacle; lbn - lower brachial nerve; ldl = lh - lophophoral dorso-lateral nerves homologous to the “lophophoral horns”; lf - latero-frontal nerve of tentacle; lg - lophophoral ganglion corresponds to central zone of the cerebral ganglion; lh - “lophophoral horns” (main nerve tract of the lophophore arm); lph - lophophore; maf - medio-abfrontal nerve of tentacle; m - mouth; mbn - main brachial nerve; mf - medio-frontal nerve of tentacle; mnr - minor nerve ring; on - outer nerve ring; tg - trunk ganglion corresponds to the proximal zone of the cerebral ganglion; tnr - tentacular nerve ring; trk - trunk; tsn - tentacle sheath nerve; tsr - tentacle sheath nerve ring

As an idea for future discussion, the zonality of the cerebral ganglion and the presence of three commissures in the cerebral ganglion of *A. gracilis* and probably of all other bryozoans might be explained as a result of merging of three ganglia (Fig. 16I). We speculate that these ganglia were located in different segments of the body in the oligomerous ancestor of bryozoans [50]. The distal zone of the bryozoan cerebral ganglion corresponds to the epistomal body part and gives rise to the “epistomal nerve ring” [41]. Because of the reduction of the epistome, the epistomal nerve ring fused with the circum-oral nerve ring that extends from the central zone of the cerebral ganglion. This zone corresponds to the ganglion of the lophophoral segment of the body (Fig. 16I). The proximal zone of the cerebral ganglion gives rise to the truncal nerves and corresponds to the ganglion of the trunk segment. Interestingly, each zone of the bryozoan cerebral ganglion has bilateral symmetry that can be regarded as resulting from the fusion of two ganglia within one body segment (Fig. 16I). The miniaturization of the bryozoans and the consequent change of their body plan might cause the fusion of the segments and their ganglia. This idea is only speculation but might be validated by future investigations and comparative analysis.

The cerebral ganglion of phylactolaemates contains a prominent lumen, which can be spacious [17, 19, 21, 40, 51] or narrow and slit like [35]. The presence of the lumen in the cerebral ganglion has been regarded as an apomorphy of phylactolaemates [35]. This idea was recently shown to be incorrect by an ultrastructural investigation of the cerebral ganglion in the ctenostome *Paludicella articulata*, which showed that this ctenostome bryozoan also has a prominent lumen in its cerebral ganglion [2]. The authors of the latter study concluded that the presence of a lumen in the ganglion is the ancestral state in the Ectoprocta. According to our data, the small cerebral ganglion of *A. gracilis* has a lumen, which occurs as a narrow space between the apical projections of the perikarya. The presence of the lumen in the cerebral ganglion of *A. gracilis* confirms the idea of Weber et al. [2] about the plesiomorphy of this state for all bryozoans.

In bryozoan ontogeny, the lumen of the cerebral ganglion forms by an invagination of the inner budding layer (epidermal layer) between the prospective mouth and anus [29, 39]. This manner of development leads to a neuroepithelial organization of the cerebral ganglion. The neuroepithelial organization of the cerebral ganglion was first described in several species of phylactomalemates [35] and more recently in the ctenostome *P. articulata* [2]. The presence of the apical adherent junctions between the cells confirms the neuroepithelial organization. In *A. gracilis*, the cells of the central zone that contact the ganglion lumen are also connected via several adherence junctions and bear basal bodies and striated rootlets. The presence of the rudimentary cilia in perikarya supports their origin from the ciliary cells. This same organization occurs in certain nerve cells in adult and larval phoronids: these nerve cells develop as a result of the submergence of ciliated epidermal cells to the basal portions of the nerve elements [52–54].

The origin of the bryozoan cerebral ganglion as a result of invagination is the annectent step in evolutionary development between the primitive intraepidermal and the progressive subepithelial nervous system. As suggested by Yurii Mamkaev [55], the collar around the tentacle nerve ring indicates that phoronids are evolving to form a more advanced subepithelial nervous system. Thus, the submerging of the intraepidermal nerve net in *Phoronopsis* spp. was regarded by Mamkaev [55] as a transitional condition between an intraepithelial nerve net and a subepithelial nervous system.

The ultrastructure of different cell types in the bryozoan cerebral ganglion is still poorly described. In many bryozoans, “giant neurons” give rise to the tentacle sheath nerves [27, 39]. In *A. gracilis*, the soma of these “giant neurons” is > 4 μm in diameter. Other perikarya are much smaller in all studied bryozoans. In the cerebral ganglion of *Asajirella gelatinosa*, some perikarya show intensive neurosecretory activity and contain numerous dense-core vesicles [32, 41]. Similar cells and their numerous projections are found in the central zone of the cerebral ganglion of *A. gracilis*.

The cerebral ganglia of the bryozoan species studied to date exhibit serotonin-, FMRFamides-, and catecholamine-like immunoreactivity [34, 37, 38, 40, 42, 56, 57]. In all cases, only neuropile neurites exhibit such immunoreactivity, whereas no immunopositive somata have been found in the bryozoan cerebral ganglion. FMRF-positive perikarya were detected only in the cerebral ganglion of *Cristatella mucedo* [57]. As reported in the current study, *A. gracilis* has a pair of serotonin-like immunoreactive perikarya associated with the ventro-lateral nerves of the cerebral ganglion. This represents the first description of serotonin-like immunoreactive perikarya associated with the cerebral ganglion in bryozoans.

### Nerves projecting from the cerebral ganglion

The main nerve originating from the cerebral ganglion is the circum-oral nerve ring. In some papers, the circum-oral nerve ring that extends from the cerebral ganglion and surrounds the mouth is called the circum-pharyngeal nerve ring [41]. The presence of one or two nerve rings running around the upper portion of the descending branch of the digestive tract is an interesting feature discussed in detail by Shunkina et al. [40]. Researchers have concluded that some bryozoans have only one ring around the mouth [58], whereas others have two nerve rings that encircle the mouth and the pharynx [20, 23, 27]. Some bryozoans have a single “circum-oral nerve ring” that is complemented by the “circum-pharyngeal nerve plexus” originating from the cerebral ganglion [2]. Such variation can be explained either by the great plasticity of the organization of the bryozoan nerve system or by the use of different microscopic techniques [40].

The circum-pharyngeal nerve ring described in some bryozoans [2, 20, 23, 27] is usually incomplete on the oral side of the pharynx [40]. In *A. gracilis*, the circum-pharyngeal nerve ring starts from the cerebral ganglion as a thick neurite bundle that extends around the pharynx and is also incomplete on the oral side.

The organization of the circum-oral nerve ring differs among bryozoans. In some bryozoans, the circum-oral nerve ring is not an entire ring due to a gap between two lateral branches on the oral side [30, 40]. Such an organization is known in phylactolaemates [19, 40], whereas most bryozoans have a complete circum-oral nerve ring [20, 22, 27, 32, 39]. In *A. gracilis*, the circum-oral nerve ring appears to be closed, but it is very thin and does not contain any serotonin-like neurites on the side opposing the cerebral ganglion.

The circum-oral nerve ring gives rise to the intertentacular (or radial) neurite bundles that pass along the intertentacular membrane to the sites between the adjacent tentacles. Their length correlates with the length of the intertentacular membrane. Radial nerves are very long in most bryozoans but are very short in *A. gracilis*. This shortness correlates with the length of the intertentacular membrane, which is almost absent in *A. gracilis*. In many bryozoans, the circum-oral nerve ring is associated with the large serotonin-like immunoreactive cells. According to a recent analysis [42], the number of serotonin-like immunoreactive cells is always equal to the number of tentacles minus two. Schwaha and Wanninger [42] have discovered the “serotonergic gap”, which is a space between the cells that are closest to the mouth and the other serotonin-like immunoreactive cells. In cheilostomates, which usually have a small lophophore with a few tentacles, there is a chain of three serotonin-like immunoreactive cells near the mouth. In phylactolaemates, which have a large lophophore, this chain includes six serotonin-like immunoreactive cells. Based on a comparative analysis, Schwaha and Wanninger [42] concluded that the anal tentacles of gymnolaemata are homologous to the lateral tentacles of the phylactolaemates, whereas the inner row of tentacles is a unique feature of the phylactolaemates. Thus, the horseshoe-shaped lophophore is the apomorphy of the phylactolamates [42].

According to our data, the localization of serotonin-like immunoreactive cells in the lophophore of *A. gracilis* cannot be explained based on the scheme proposed by Schwaha and Wanninger [42]. Our investigations revealed that there is the “serotonergic gap” in *A. gracilis* between the two serotonin-like immunoreactive cells that are near the anus and the six other serotonin-like immunoreactive cells. Such location of serotonin-like immunoreactive cells seems to be different from the scheme described for the closest species *Amathia semiconvoluta* [42]. In this species, there are three analmost and three oralmost serotonin-like immunoreactive cells. Thus, two closely related species have different organization of the serotonin-like immunoreactive nervous system; that bears evidence about plasticity in bryozoan neural patterning.

At the same time, *A. gracilis* has a pair of special serotonin-like immunoreactive cells that are located adjacent to the anus and that are associated with two dorso-lateral nerves of the lophophore. These nerves start from the cerebral ganglion and participate in the innervation of the four tentacles that are closest to the anus. According to their position, the lophophoral dorso-lateral nerves of the ctenostome *A. gracilis* (Fig. 16IIc) correspond to the “horns of the lophophore” (dorso-lateral horns of the cerebral ganglion) typical for most phylactolaemates with the horseshoe-shaped lophophore (Fig. 16IIa); these lophophoral dorso-lateral nerves are present even in species with the bell-like lophophore, e.g., *Fredericella sultana* (Fig. 16IIb) [35, 40]. Interestingly, all bryozoans have one pair of “border” (or “frontier”) tentacles innervated by both nerve tracts: “the horns of the lophophore” (the lophophoral dorso-lateral nerves in *A. gracilis*) and the circum-oral nerve ring (Fig. 16II). The location of these “border” tentacles probably corresponds with the “serotonergic gap” described by Schwaha and Wanninger [42].

The presence of the “lophophoral horns” in ctenostome bryozoans once again raises the question about the nature of the ancestral pattern of the bryozoan lophophore. Our data suggest that the ancestral type of the bryozoan lophophore is represented by the horseshoe-shaped lophophore characteristic of the large ancestral species and retained by recent phylactolaemates (Fig. 16II).

However, another scenario is also possible. As the presence of perikarya in dorso-lateral nerves of the lophophore of *A. gracilis* is not definitely established, these nerves seem to be quite different from the “horns of the lophophore” containing many perikarya in phylactolaemata. Moreover, in phylactolaemata, the “horns of the lophophore” give rise to the medio-frontal nerves of tentacles, whereas dorso-lateral nerves of the lophophore of *A. gracilis* do not give rise to the medio-frontal nerves of tentacles. In this light, the presence of dorso-lateral nerves of the lophophore in *A. gracilis* might be regarded as ancestral condition, which evolved further and gave rise to the complex and large “horns of the lophophore”. In such scenario, the simple lophophore in bryozoans is the most primitive state, whereas horseshoe-shape lophophore of phylactolaemates is the secondary state.

The outer nerve ring in *A. gracilis* is here described for the first time in an ectoproct. This neurite bundle passes around the abfrontal side of the tentacles: such a position corresponds to the outer lophophore nerves in other lophophorates (Fig. 16III). Thus, both phoronids and brachiopods have an outer lophophoral nerve: the tentacular nerve ring in phoronids (Fig. 16IIIa) and the lower brachial nerve in brachiopods (Fig. 16IIIb). In both groups, the outer nerve of the lophophore gives rise to the abfrontal neurite bundles of the tentacles. In *A. gracilis* and other bryozoans, the abfrontal neurite bundles originate from the inner nerve ring (circum-oral nerve ring) or from a part of the ring-the intertentacular nerves-while the outer nerve ring does not participate in tentacle innervation. However, the presence of the outer lophophore nerve in bryozoans, phoronids, and brachiopods makes the neuronal architecture of the lophophore extremely similar in all three groups. This similarity supports the homology of the lophophores and the monophyly of the lophophorates [11].

### Innervation of the tentacles

Bryozoans exhibit several patterns of tentacle innervation. The organization of the tentacle innervation is the most complex in some phylactolaemates [40]. Most phylactolaemates have six basiepidermal tentacular nerves: medio-frontal, medio-abfrontal, one pair of latero-abfrontal, and one pair of latero-frontal. Gymnolaemates in general have four tentacular nerves: one abfrontal, one medio-frontal, and one pair of latero-frontal [2, 39]. Thus, in general, bryozoans possess four to six longitudinal neurite bundles: one medio-frontal nerve, one abfrontal nerve, and one pair of the latero-frontal nerves. New data on innervations of tentacles in ctenostome bryozoans allowed to conclude that presence of four tentacle nerves are the ancestral state in Ectoprocta and not six as proposed earlier [2].

According to our results, *A. gracilis* has only two tentacular nerves. The fusion of the latero-frontal and the medio-frontal neurites taking place at the tentacle base, results in a single frontal tentacular nerve. This type of the tentacles innervations is unique among all ectoprocts. The presence of only two nerves in each tentacle of *A. gracilis* might be regarded either as a result of reduction in the number of tentacular nerves or as ancestral condition. In the first case, the presence of six tentacular nerves should be regarded as ancestral condition for all bryozoans. In the second case, the presence of six tentacular nerves should be the most advanced condition. The first scenario supports an idea about progressive miniaturization of bryozoans and simplification of the lophophore shape. The second scenario supports an idea about the increasing of the bryozoan’s body and development of the more complex lophophore.

Six tentacular nerves are characteristic for all other lophophorates: phoronids and brachiopods (Fig. 16III) [11, 59]. The innervations of tentacles together with the similar gross anatomy of the lophophore nervous system is additional evidence of lophophorates monophyly.

In addition to six or four basiepidermal nerves, many ectoprocts have two basiperitoneal tentacular nerves [32]. In *A. gracilis*, there are at least two basiperitoneal nerves in each tentacle. Each basiperitoneal tentacular nerve is represented by a single neurite of large diameter that extends along the lateral side of each tentacle. Such a neurite has electron-lucent cytoplasm containing thick microtubules. According to some data [2], these neurites do not show immunoreactivity against acetylated α-tubulin and thereby cannot be regarded as nerve elements. Similar neurites with specific ultrastructure occur in the tentacles of phoronids [60] and brachiopods [11, 61]. In arcitulate brachiopods, these neurites are numerous and exhibit strong immunoreactivity against acetylated α-tubulin [61]. The presence of such an unusual element as basiperitoneal neurites makes the innervation of tentacles in all lophophorates quite similar and together with other common features of the lophophore neuroarchitecture suggest the homology of the lophophore.

Based on their origin, two main types of tentacular nerves occur in bryozoans. The first type is common in phylactolaemates and is characterized by the branching of all tentacular nerves from intertentacular (radial) nerves [40]. The second type occurs in gymnolaemata in which some nerves originate from intertentacular nerves and others originate directly from the circum-oral nerve ring [2, 23, 39]. There are some variations in the second type. For example, in most of the gymnolaemates, the medio-frontal nerves arise from the circum-oral nerve ring while the abfrontal and the latero-frontal nerves branch from the intertentacular nerves. In *E. pilosa*, in contrast, the abfrontal nerve also originates from the circum-oral nerve ring [23].

In all cases mentioned above, the abfrontal nerve results from the fusion of two or more neurite bundles that arise from two adjacent intertentacular nerves or directly from the circum-oral nerve ring [2, 23, 39]. *Electra pilosa* and *A. gracilis* have a unique type of tentacle innervation: the abfrontal nerve originates as a single neurite bundle.

## Conclusion

A detailed investigation of the lophophore innervation in ctenostome bryozoans revealed some intriguing features that increase our understanding of the lophophore and the organization of the body of ancestral ectoprocts. First, the zonality of the cerebral ganglion, the presence of three commissures, and the location of main nerves emanating from each zone may have resulted from the fusion of three different ganglia in three different body parts in the bryozoan ancestor: epistomal, lophophoral, and truncal. On the other hand, this zonality might result from the directive innervation of the various parts of the body. Second, the presence of a lumen in the cerebral ganglion and its neuroepithelial nature represent the ancestral features of the nervous system in bryozoans. Third, the dorso-lateral nerves in the *A. gracilis* lophophore might correspond to the greatly developed “lophophore horns” of the horseshoe-shaped lophophores in the phylactolaemates. Thus, the large horseshoe-shaped lophophore might be regarded as the ancestral type of the lophophore in the bryozoans. Contrary scenario is also possible. Fourth, the presence of the outer nerve ring, which corresponds to the lower brachial nerve of brachiopods and to the tentacular nerve ring of phoronids, makes the gross neuronal morphology of the lophophore very similar among bryozoans, brachiopods, and phoronids. Fifth, the existence of the six nerves within each tentacle is the ancestral feature for all bryozoans. All other types of tentacle innervation should be regarded as a result of the reduction due to the decrease in lophophore size and in tentacle number. Sixth, the location of the main nerves and the tentacle innervation suggests the homology of the lophophore in bryozoans, phoronids, and brachiopods, and is consistent with the inference that the lophophorates are monophyletic.

## Additional file

Additional file 1: Running projection including volume rendering of the anti-acetylated tubulin staining (grey) and 3D reconstruction of serotonin-like nervous system. The projection starts from the oral side and ends at anal side. Large seroronin-like immunoreactive perikarya (green) are located between the tentacles bases and connect to the circumoral nerve ring (blue). (AVI 48362 kb)
